# Exploring canine olfactory generalization using odor profile fractions from native crude oils

**DOI:** 10.1371/journal.pone.0311818

**Published:** 2024-10-17

**Authors:** Michelle Karpinsky, Daniel Lopez, Erik Campues, Paul Bunker, Stephanie R. Vaughan, Howard K. Holness, Kenneth G. Furton, Lauryn E. DeGreeff

**Affiliations:** 1 Department of Chemistry and Biochemistry and the Global Forensics and Justice Center, Florida International University, Miami, Florida, United States of America; 2 Chiron K9, San Antonio, Texas, United States of America; BOKU: Universitat fur Bodenkultur Wien, AUSTRIA

## Abstract

Canines are used by both government agencies and industries for their keen olfactory capability as well as selectivity, reliability, versatility, and speed. Within the last decade, canines have been used for the detection of on-shore crude oil. They were previously shown to find these deposits with high accuracy, providing increased confidence with little risk to oil spill response survey teams. In order to efficiently train canines, it is important to understand the odorants or groups of odorants that such canines use when locating subsurface crude oil deposits, as well as track how the odorant profile changes as the crude oil undergoes degradation. In this study, headspace solid phase microextraction (HS-SPME) was used in combination with gas chromatography-mass spectrometry (GC-MS) to extract and separate odorants from the headspace of various crude oils. After, eluent fractions of the crude oil odor profile were separated and collected onto sorbent materials, which were then used as canine testing probes in a series of trials. These probes, along with negative and positive controls were presented to three previously-trained and operational crude oil detection canines. Three eluent fractions of both fresh and weathered samples were presented, resulting in a 100% response rate from the canines on all three fractions from both the fresh and weathered samples. These results indicated that canines are capable of detecting crude oil from any fraction of the odor profile demonstrating the potential of the canines to generalize across a variety of crude oils and stages of weathering.

## Introduction

A canine’s olfactory system is highly selective and sensitive, leading them to be used as detectors in many fields, such as in law enforcement for the detection of narcotics [1] and explosives [2], medicine for the detection of diseases [3], search and rescue for the location of human scent (living [4] and deceased [5]), and environmental for the location of invasive or endangered species [6] or for the detection of oil spills [7]. Though canines are exceptional at finding target odorant(s), challenges may arise in the training of canines to detect complex mixtures.

Canines are highly capable at generalizing between like odor profiles of similar target odors, while also being able to discriminate these profiles from outside factors such as background odors, and other non-target substances that have a similar odor make-up. Generalization refers to a canine’s ability to respond to an odor with similar make-up to that of a target odor while ignoring subtle differences between them [8]. Operationally, related target odors will come from an array of origins, variants, and purities that the canine must generalize between [9]. Conversely, with discrimination a canine will recognize the subtle differences between two similar stimuli, classify each under a different categories, and respond differently [8]. Discrimination is important when training a canine so that they may ignore background and distractors in the field as well as ignore substances containing odorants common to the trained target odor. For example, cocaine and snapdragon flowers share a common odorant of methyl benzoate. Canines trained to alert to methyl benzoate for cocaine detection need to generalize between different variants and manufactured cocaine, while simultaneously discriminating against snapdragons. Cerreta et al. demonstrated trained drug detection canines correctly detected to cocaine while ignoring the snapdragons [10]. Canines have also been shown to generalize and discriminate between structurally similar compounds [9]. Hall et al. investigated the performance of domestic dogs and their ability to discriminate against homologous alcohols. They found dogs were able to discriminate against alcohols, differing in the number of carbons, to their target odorant of 1-pentanol [11].

Generalization and discrimination become more complicated when considering complex mixtures. Moser et al. defined elemental processing as the perception of individual components of the mixtures separately and configural processing as the perception of the mixture as a unique odor, giving the mixture a different odor quality and not just the sum of its component odorants [8]. One can consider a mixture composed of beef stew, where elemental processing would be the identification of the individual ingredients (beef, carrots, potatoes, etc.), and configural processing would be perception of the stew as a whole [12]. Canines have been observed to do exhibit both elemental and configural processing under different circumstances, and the processing method can lead to either generalization or discrimination. To date, research has focused on either single odors or simple mixtures, such as with explosives, to provide a better understanding of how canines generalize and discriminate to target odors. For example, Lazarowski et al. found that canines trained only on ammonium nitrate (AN) [13] and pure potassium chloride (PC) [14] had difficulty generalizing to realistic AN-based and PC-based targets. While DeGreeff et al. demonstrated that canines trained to detect AN alone generalized to AN-based mixtures [15]; however, both researchers noted the tendency to generalize or discriminate from a trained odor to that odor in a mixture was partially dependent on the individual animal’s perception and odor processing. The different studies suggest that there may be other influences, such as training procedure and odor delivery methods, that affect the way a canine detects odor within a mixture.

The aforementioned studies on generalization and discrimination were conducted on materials where the main odorants had been previously known, like cocaine and methyl benzoate and the main odorants in some explosives like AN and PC; however, for other detection targets, like human scent and other biological or living specimens, the target odors are complex odorant mixtures where the specific components are not necessarily known [8] complicating canine generalization and discrimination training. Oldenburg et al. sought to train wildlife detection dogs to generalize between odor variations of otter feces, while retaining specificity against other mammals’ scat. The canines were trained to generalize between fresh to 2-week-old scat, as well as on scat from otters of different diets (captive and wild). Though only one canine was used, the researchers found that she was able to detect the different variations of otter scat from only being trained on captive otter scat, while successfully ignoring other mammal scat [16]. DeChant et al. [17] studied oil-specific discrimination in detector dogs to determine whether dogs trained on fresh crude oil could successfully discriminate against weathered oils for the purpose of assisting in oil response on shorelines. Three canines were trained on a fresh sample and tested on the fresh and four weathered oil samples resulting in the dogs ignoring the weathered samples and alerting to the fresh samples. The proof-of-concept study demonstrated that dogs could be readily trained to alert to a fresh oil sample and ignore tarballs and weathered oil.

It is possible to determine the key odorants that are responsible for a canine alert by presenting a canine previously trained to detect the target odor as a whole with individual odorants associated with that target [1]; however, for complex odorant mixtures, such as crude oils, this approach is not feasible. One possible way to assess what canines detect within complex mixtures is to divide the odorant profile into fractions by use of gas chromatography. Fuller et al. was one of the first to use this method of separation through gas chromatography–olfactometry (GC-O) for a human olfactory study where the chemical odorants associated with a perfume were separated and delivered to a professional perfumiest to detect and describe the odorants as the eluted from the GC [18, 19]. GC-O is currently applied to human assessors exclusively, as training a canine to sit and sample over the duration of a GC-O run, which can be as long as 30 minutes or more, is highly challenging [19]. Instead, GC-fractionation (GC-F) allows for the same principle of GC-O, separating odor profiles through a GC, followed by collection of the GC eluent onto a sorbent material to then later be presented to a non-human assessor, such as a canine. A proof-of-concept study completed in 2017 demonstrated the use of odor fractions to identify the active odorants of the invasive fungus, *Raffaelea lauricola*. Here the authors identified nine common odorants between avocado trees infected with the *R*. *lauricola* phytopathogen, and used the knowledge to create four, 10-minute fractions from the overall profile to determine the target odorants associated with the infected trees [20]. Two additional studies used GC-F to probe odorants of interest to trained detection canines for other highly complex mixtures of volatile organic compounds (VOCs), human scent and crude oil, respectively [21, 22]. Where Simon et al. collected and separated nine target odorants, which were presented to canines, Hudson et al. and Vaughan et al. collected fractions of the complex odor profile onto sorbent materials and presented those fractions in a series of controlled studies. Vaughan et al. [22] completed a preliminary testing session using two canines, previously trained to detect crude oils from differing origins and conditions, to determine the components used by oil detection canines to significantly improve training procedures for these dogs. The canines were presented with three fractions from the fresh crude oil and alerted 67% to two of the three fractions and 100% to the other fraction. The small study demonstrated a canine’s capability to detect crude oil using any fraction of the profile.

This work endeavored to replicate and build upon the preliminary work of Vaughan et al. [22] in a more robust study and further explore the limitations of detection canines. The use of canines to detect crude oil originated in the late 2000’s in Norway as a method to detect spilled crude oil in artic regions. Formal and informal studies were completed from 2008 to 2009 by SINTEF (Norwegian: Stiftelsen for Industriell og Tenknisk Forskning) scientists and Trondheim Dog Training Academy trainers, verifying the use of canines to detect subsurface oil residues in beach sediments or under snow and ice [23]. Other studies were conducted on canines detecting subsurface oil in locations such as the North Saskatchewan River [24], Prince William Sound, Alaska [25], and Somerset, Texas where canines demonstrated the ability to detect crude oil pits as deep as 15 ft (5 m) buried under the surface [26, 27]. These studies showed that canines were able to find subsurface crude oil with high accuracy, providing confidence for including canines in survey teams. The inclusion of canines to survey teams would increase assessment speed, reduce labor of intensive processes such as excavation and coring, and provide accurate and reliable locating of subsurface oil pits [27]. For field operations, there may be a desire to train canines to generalize between differently sourced crude oils, while simultaneously discriminating between fresh and weathered crude oil. In this study, eluent odor fractions were made from fresh and weathered crude oil and were presented to canines over the course of three trials. The aim of this study was to determine the odorants or groups of odorants that canines used when locating subsurface crude oil based on the responses of the dogs to each fraction. The knowledge gained may provide a better understanding of a canine’s recognition to complex odors as well as to improve training aids by applying the end results to real world applications.

## Materials and methods

### Overview

The study used headspace solid phase microextraction (HS-SPME) and gas chromatography-mass spectrometry (GC-MS) to analyze the headspace of fresh and weathered crude oils. Once the vapor profile of each crude oil sample was established by HS-SPME and GC-MS, fractions of the GC eluent were collected by removing the GC column from the MS detector and threading into the back inlet of the GC allowing the eluent to be collected onto a substrate. The collected fractions were provided for canine testing. All procedures regarding canine trials were approved by the Florida International University Institutional Animal Cares and Use Committee (Protocol #201614). The trials were conducted by professional handlers both trained and certified in the care and ethical use of canines. Additionally, odor from the fractions were confirmed by secondary collection of VOCs emanating from the fractions again by SPME-GC-MS.

### Materials

Three crude oils were used throughout the study, including fresh West Texas Intermediate (WTI) crude oil, provided by Naval Research Laboratory (NRL, Washington, DC) and Bureau of Safety and Environmental Enforcement (BSEE, Washington, DC), as well as fresh Hibernia crude oil and 20% photo-oxidized Hibernia crude oil, provided by the Oil Spill Response Research and Renewable Energy Test Facility (Ohmsett, Leonardo, NJ). Fresh WTI crude oil was an oil harvested off of the Gulf Coast. It is a light, sweet crude, meaning it has a higher American Petroleum Institute (API) gravity, is less viscous, and is used to produce gasoline and diesel products, while also having a low sulfur content producing a sweeter smell. According to NRL and BSEE, the WTI crude oil sample received was fresh and kept within the reserves stored under ambient pressure and temperature. The samples were received at Florida International University (FIU) in 2021 and stored in a refrigerator at 4 °C until sampling. Hibernia crude oil was harvested off of the coast of Nova Scotia, located in the Atlantic Ocean. Hibernia crude is a light, sweet crude oil. The fresh Hibernia crude oil had been stored within the oil reserves housed by Ohmsett until it was received by FIU where it was then refrigerated at 4 °C until sampling. Fresh Hibernia crude oil was photo-oxidized until 20% mass weight loss at Ohmsett, collected, and shipped to FIU where it was refrigerated at 4 °C until required for analysis.

### Instrumental analysis

The extraction of the headspace of crude oil utilizing SPME was developed by Vaughan et al [22] and used for this study. For this purpose, 1 mL of crude oil was placed into a 20 mL VOA vial, which was set into an aluminum block heated at 35 °C. The crude oil was allowed to equilibrate for 20 min, before extraction with a 100 μm polydimethyl siloxane (PDMS) SPME fiber (Supelco) for 10 min. Following extraction, the SPME fiber was thermally desorbed in a GC inlet at 250 °C for 4 min. All fibers were cleaned after use by heating the fiber in a GC inlet at 250 °C for 4 min to allow for any remaining compounds to desorb off of the fiber before being reused.

The GC-MS method was adapted from Vaughan et al. [22] and the parameters are reported in Table 1. For this purpose an Agilent 8890 GC with 5977B MSD was utilized with an RTX-Volatiles column (Restek, 30 m × 0.25 mm I.D. × 1.0 μm).Compounds were tentatively identified by comparison matching to those compounds in the NIST mass spectra library (version NIST20) as well as to two alkane ladders, C6-C10 (AccuStandard) and C11-C18 SPEX CertiPrep), and BTEX standards (SpexCertiPrep).

**Table 1:** GC-MS parameters.

*Method Parameters*	
*Inlet mode*	Temperature: 250 °C 10:1 split 2 mL/min helium flow rate
*Oven program*	35 °C, hold 1 min 35–80 °C at 8 °C/min 80–200 °C at 5 °C/min, hold 2 min 200–250 °C at 30 °C/min
*Mass Spec Parameters*	Transfer line at 250 °C Mass range (m/z 30 to 450)

### VOC fraction collection

**Fig 1:**
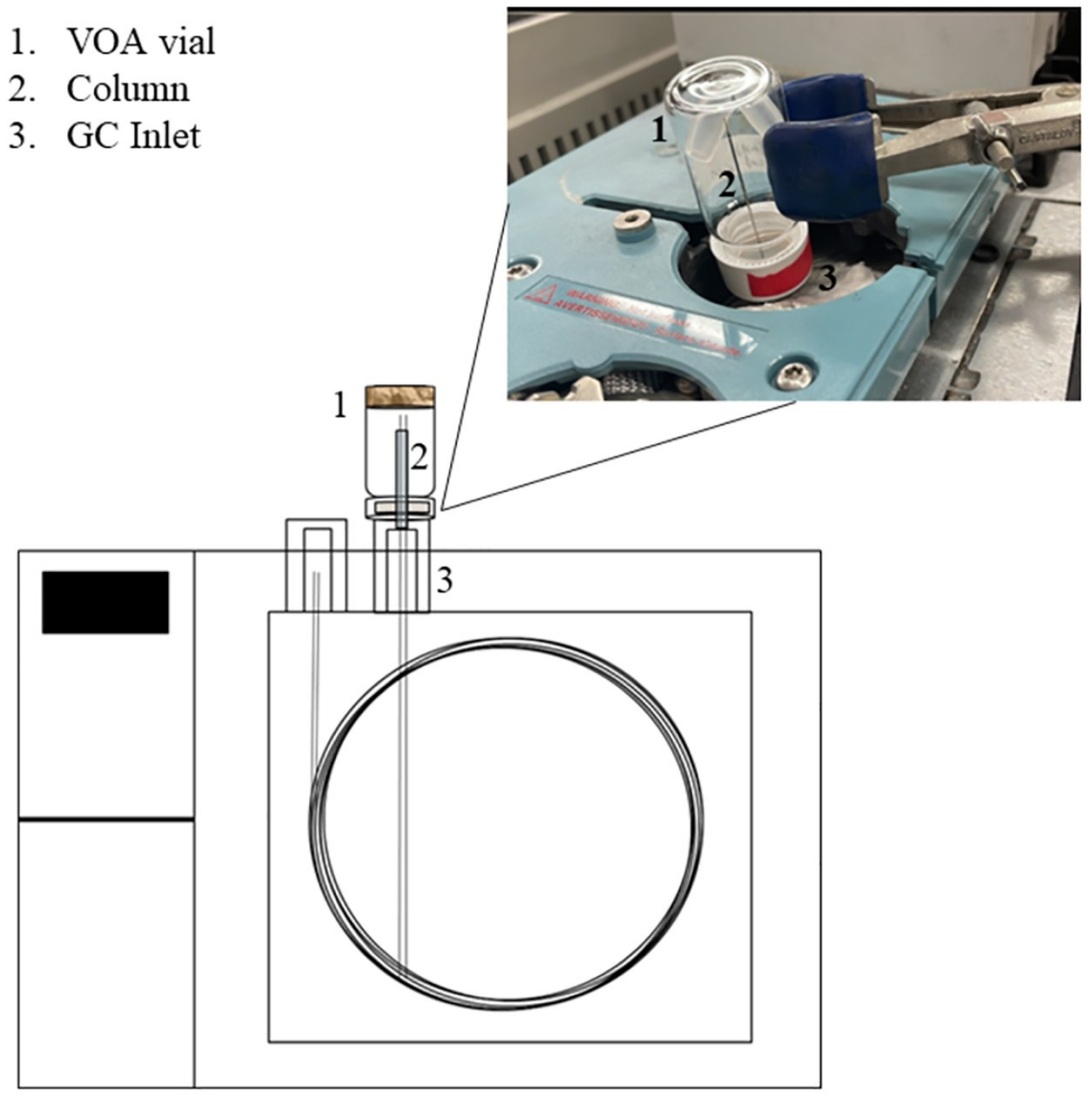
Diagram of the gas chromatogram fractionation with a window showing the actual instrument.

Crude oil headspace fractions was completed using a separate 6890 Agilent GC, as depicted in Fig 1. The end of the column was removed from the detector and threaded through the unused back inlet of the GC allowing the eluent to vent to the atmosphere. A needle from a gas tight syringe was placed over the exposed end of the column with a septum holding it in place, which was then used to pierce through the cap of a VOA vial containing the sorbent material. For this study, Whatman 1 cellulose filter paper (55 mm diameter) was used as the sorbent material. Previous studies on sterilized gauze determined that it was not “analytically clean” and could contain VOCs of interest [28, 29] so to ensure no additional odors were present, the filter paper was baked in an oven at 105 °C for an hour. Fractions were collected based on the volatility of the odor profile: highly volatile (retention times [t_r_] 0 to 7 min), volatile (t_r_ 7.1 to 15 min), and semi-volatile (t_r_ 15.1 to 34.29 min). Fraction samples were collected twice onto a single sorbent to ensure sufficient odor was collected onto the sorbent material for detection by canines. A clean method was run between collection of each fraction to avoid any cross contamination from occurring between samples. Along with the fractions, blanks, negative controls, distractors, and positive controls (Table 2) were also collected using this method. The elution collection oven parameters for the 6890 GC were the same as the method described above in Table 1. The inlet parameters were changed from a 10:1 split to splitless.

**Table 2:** Samples used throughout all three trials.

SAMPLE NAME	SAMPLE DESCRIPTION
**CONTROLS**	
POSITIVE CONTROL 1	Entire crude oil odor profile
POSITIVE CONTROL 2	Odor soak of crude oil
BLANKS	Odor collection from the GC column
TRIP BLANK	Clean filter paper placed into vials to collect odors present during shipping
DISTRACTORS	Peanut butter, nitrile gloves, packing foam, sharpie, isoamyl salicylate, and cologne
SPME FIBER	SPME fiber odor
**TESTING PROBES**	
FRACTION 0	Initial fraction from rt 0–2.5 min of the fresh WTI crude oil
FRACTION 1	Highly volatile fraction from rt 0–7 min
FRACTION 2	Volatiles fraction from rt 7.1–15 min. In background threshold study, rt changed to 2.51–24.59 min
FRACTION 3	Semi-volatiles fraction from rt 15.1–34.29 min
FRACTION 3.5	End fraction from rt 30–34.29 min of the fresh WTI crude oil
CRUDE OIL WITH SINGLE ODOR	Entire WTI crude oil odor profile sampled with isoamyl salicylate
CRUDE OIL WITH COMPLEX ODOR	Entire WTI crude oil odor profile sampled with cologne

To ensure that eluent VOCs were collected by the GC-F method, the headspace of an additional set of odor fractions collected onto filter paper were sampled and analyzed using the above SPME-GC-MS method; however in this case, the filter paper was equilibrated in an aluminum block at 35 °C for 24 hours and the headspace was extracted for 1 hour. The 10:1 split on the GC-MS method was changed to splitless due to their being less odor absorbed on the fiber from the recollection.

### Canine testing

Samples collected by the GC-F method included blank/negative controls, distractors, positive controls, and testing probes (Table 2). The blanks/ negative control include blank column runs from the GC column and trip blanks. The blank column runs were collections of the eluent from the GC column onto filter papers when no sample was introduced into the column. Trip blanks were filter paper placed into VOA vials and transported to the field. These served to ensure that samples were not contaminated throughout the sampling and handling process before the trials. Distractors were non-target odors, such as food or other commercial products. For this study the chosen distractors odors included sharpies, peanut butter, nitrile gloves, and packaging foam. Positive controls were a collection of the entire odor profile from the crude oils, in other words the entire chromatogram eluent was collected on to a single substrate. Positive controls were used intermittently during the trials to reinforce canines and confirm they were producing a reliable alert and properly trained. The testing probes consisted of the fractions made during the study. GC-F samples were collected and coded prior to being distributed to the testing personnel. After collection and for storage the samples were packaged into small mylar bags (Amazon, 4”x6”), which were then packaged into large barrier blocking foil pouches (TED PELLA INC, large, 8x10-1/2x3-1/2”) to eliminate cross-contamination between samples. For shipping, all samples were cold packed to ensure the odor stayed present through shipping, and shipped to Chiron K9 (San Antonio, TX) where the canine trials took place.

All canine testing was conducted in a double-blind fashion, meaning neither the canine handler nor the test assessor/administrator was aware of the identity of the samples. An assistant set up all the trials using the identification key provided by FIU but was removed from the canine testing space. Results were returned to FIU to determine correct or incorrect canine response. The assistant was in a control room with mirrored glass windows. The handler was not able to see the assistant, but the assistant was able to view the trials. If the canine indicated correctly on a control or known source the assistant would indicate with a green light so the handler could reward the dog.

Canine search motivation was maintained by inclusion of a known training aid odor in the testing. For this purpose, the substrate was placed directly in the headspace of the crude oil, allowing for collection of the odor profile without use of the GC-F. These samples were presented in a warm-up round before the trial began. After the warm-up round, the trial proceeded with six double-blind rounds, which held the sample probes and control samples. The test administrator periodically included additional known odor samples to maintain motivation. No data was collected from the warm-up round. In addition to the warm-up round, blank rounds were included in the trials were no targets where presented. Canines were trained to give a response, different from the response when a target was detected, when no odor was present and would give an “all clear” if they found no odor in the line-up. If no target odor was detected, the canine would leave the set-up and return to the handler. This provided the canine an option when or if a target odor was not detected, reducing the risk of false positive responses as well as prevent the canines from becoming frustrated as an “all clear” was a rewardable behavior.

To ensure that there was no contamination between rounds, the stands holding the samples were cleaned before and after each round. The vials were removed using disposable rubber gloves, different gloves for each vial, and the stands were wiped down with acetone on which was on a sterile gauze. The stands were left to air dry for five minutes, and then a canine, trained in hydrocarbon detection, was used to search the stands and ensure no oil contamination was residually present. If there was no canine response, then the stands could be set up for the next set of samples. This was done throughout the entirety of the three trials.

Three canines were used in total. Two of the canines, Poppy and Nika, were generic oil detection dogs, canines trained to detect both fresh and weathered oil, both with field deployed experience and thousands of verified finds during their deployment. The third canine, Luna, was a specific oil detection canine, only trained on fresher samples of WTI crude and Bunker C crude. Luna had no deployment experience, except training along Texas beaches. All canines had previous experience and training with the particular testing set up used herein. Poppy and Nika participated in all three trials, while Luna only participated in the first two trials.

## Results

### Fresh crude oil

**Fig 2:**
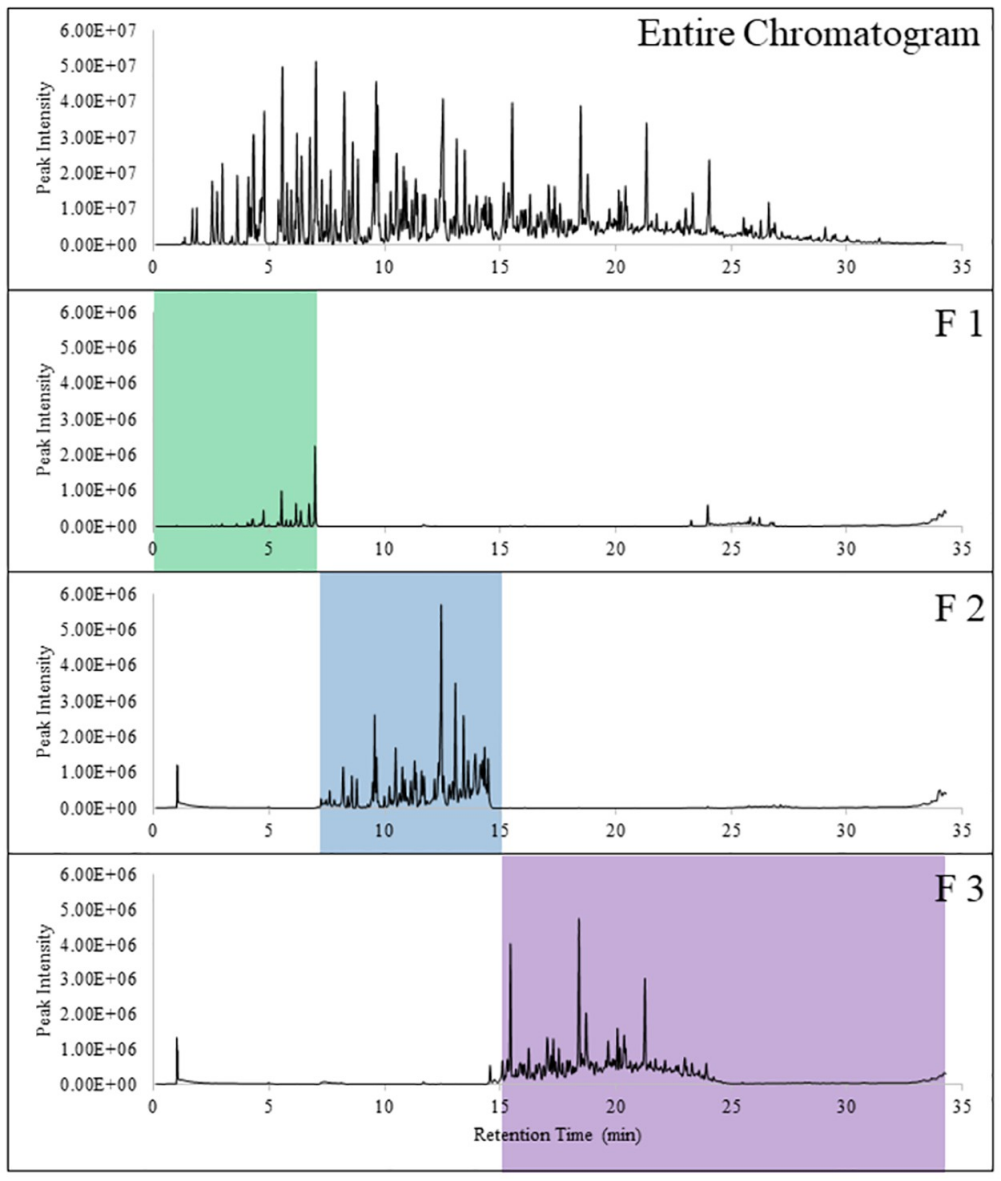
Fresh WTI crude oil entire chromatogram and its corresponding fractions. F1 was the highly volatile fraction collected between 0 to 7 min, F2 was the volatile fraction collected between 7.1 min to 15 min, and F3 was the semi-volatile fraction collected between 15.1 min to 34.29 min.

The canines were tested for their ability to recognize fractions from two different sources of fresh crude oil. Fig 2 shows the entire chromatogram obtained directly from the fresh WTI crude oil and analyzed using GC-MS and S1 Table lists the compounds tentatively identified in the headspace. To collect the three fractions, also shown in Fig 2, the chromatograph eluent, such as seen in Fig 2 (top), was collected onto a filter paper substrate, then extracted from the substrate, and analyzed using GC-MS. The chromatograph below shows the resulting compounds found in the headspace from each of those fractions. Fraction 2, the volatile fraction, and Fraction 3, the semi-volatile fraction, contained more compounds, both in quantity and abundance, than what was recovered in Fraction 1, the highly volatile fraction. Fraction 1 contained compounds that have low boiling points, that may have led to some of those compounds being lost over a period of time. Comparing the fractions to the entire chromatogram, the fractions contain an order of magnitude less in abundance. The decrease in abundance may have been as a result of the amount being absorbed onto the filter paper being less, loss during the collection method, and/or the compounds remained on the paper during re-extraction. Fraction 1 was also missing compounds between 1 and 3 minutes, which may have been as a result of a loss of compounds during collection or due to the recollection method.

“Results from canine testing with the fresh crude oil sessions are provided in Tables 3 and 4 for fresh WTI crude oil and fresh Hibernia crude oil, respectively.” Three canines were tested for their ability to alert to both fresh crude oils. The fresh Texas crude oil fractions were presented a two times and the positive control was presented once. All three canines alerted to the positive controls and each of the three fractions from the fresh WTI crude oil. Nika had one false response when she hesitantly responded to a negative before moving on and alerting on the fraction. The same negative control was later presented during the blank round of the session and Nika did not alert. No other false alerts occurred on the WTI fractions. The fresh Hibernia crude oil fractions were presented one time, once for each fraction, and the positive control was presented two times. All three canines alerted to the positive controls, and, again, the canines alerted to all three of the fractions 100% of the time. Luna had one false alert on a distractor (cologne). The distractor was presented again in a later round, and Luna did not alert to that distractor. The results indicated the canines are able to use parts of the odor profile for detection of crude oil, despite the source of oil.

**Table 3:** Number of correct responses to fresh WTI crude oil, positive control and fractions, by each canine. An alert is represented by A and no alert/ response is represented by N. Within this session, 41 negative controls and distractors were presented. n represents the number of times a sample was presented throughout the trial.

	Fresh West Texas Intermediate Crude Oil				
	Positive Control (n = 1)	F 1 (n = 2)	F 2 (n = 2)	F 3 (n = 2)	False Response (n = 41)
**Poppy**	A	A	A	A	0
**Nika**	A	A	A	A	1
**Luna**	A	A	A	A	0
**Total Response**	**3**	**6**	**6**	**6**	**1 out of 41**
**Percent Alert**	**100%**	**100%**	**100%**	**100%**	**2.44%**

**Table 4:** Number of correct responses to fresh Hibernia crude oil, positive control and fractions, by each canine. An alert is represented by A and no alert/ response is represented by N. Within this session, 31 negative controls and distractors were presented. n represents the number of times a sample was presented throughout the trial.

	Fresh Hibernia Crude Oil				
	Positive Control (n = 2)	F 1 (n = 1)	F 2 (n = 1)	F 3 (n = 1)	False Response (n = 31)
**Poppy**	A	A	A	A	0
**Nika**	A	A	A	A	0
**Luna**	A	A	A	A	1*
**Total Response**	**6**	**3**	**3**	**3**	**1 out of 31**
**Percent Alert**	**100%**	**100%**	**100%**	**100%**	**3.23%**

* Note: Canine alerted on new distractor (cologne).

### Weathered crude oil studies

**Fig 3:**
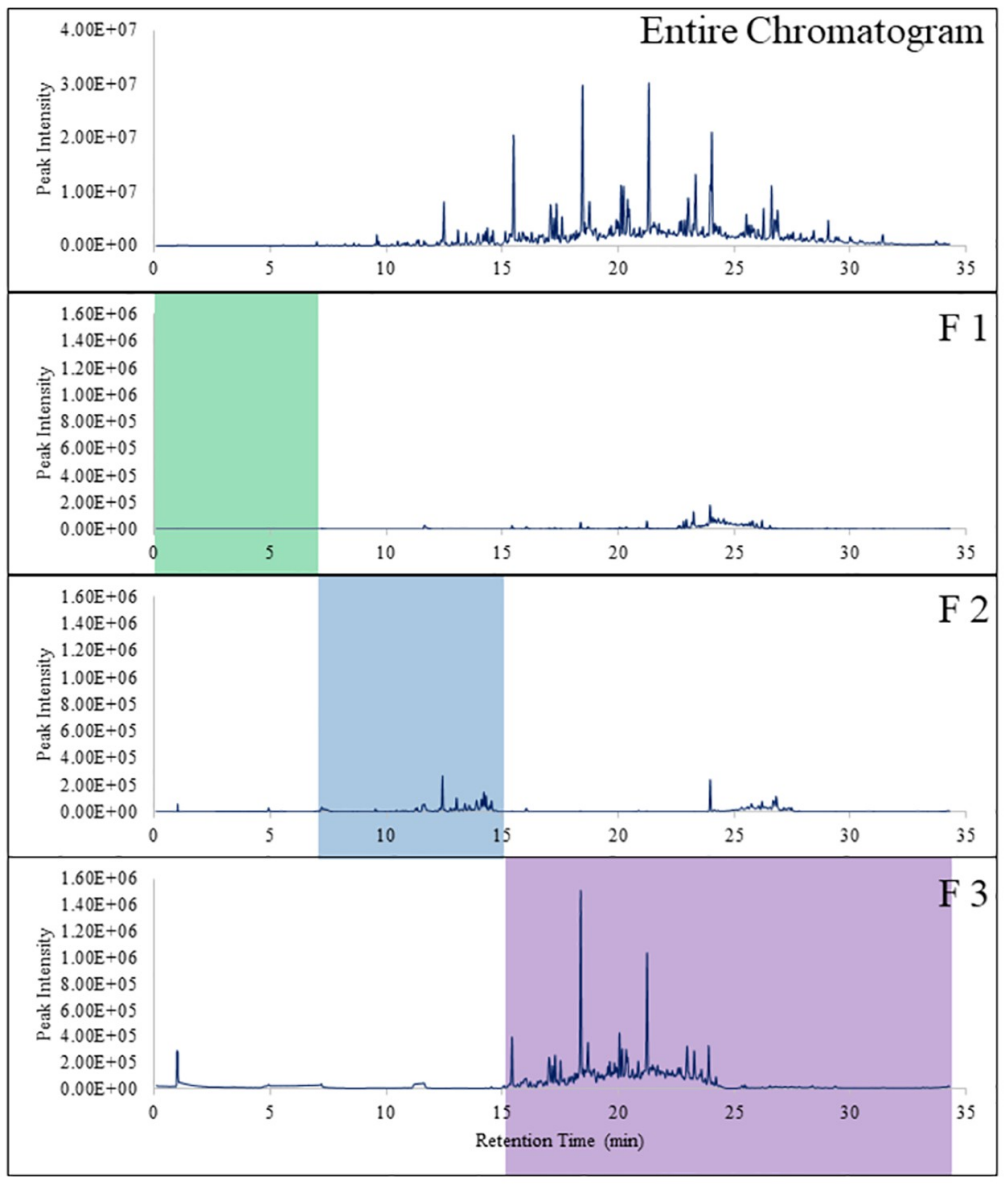
20% Photo-oxidized Hibernia crude oil entire chromatogram and its corresponding fractions. F1 was the highly volatile fraction collected between 0 to 7 min, F2 was the volatile fraction collected between 7.1 min to 15 min, and F3 was the semi-volatile fraction collected between 15.1 min to 34.29 min.

Three canines were tested for their ability to alert to a weathered sample of Hibernia crude oil. Fig 3 displays the resulting entire chromatogram, and the three fractions of the 20% photo-oxidized oil and S2 Table lists the compounds tentatively identified in the headspace. The weathered sample had little to no compounds present within Fraction 1. The loss in VOCs was attributed to those compounds being present below the limit of detection of the instrument or as a result of the weathering the crude oil had undergone. Fraction 3 contained the most odor and abundant compounds comparatively to Fraction 1 and 2 as semi-volatiles compounds are the most prevalent compounds after the crude oil has undergone weathering.

Results from the canine tests with the weathered crude oil are provided in Table 5. Three canines were tested on the weathered Hibernia crude oil. The fractions were presented one time, once for each fraction, and the positive control was presented four times. The canines alerted to all three fractions of the 20% photo-oxidized Hibernia crude oil. One false alerts was recorded for Luna; however, the other two canines did not alert on the distractors and negative controls. The false alert occurred during the presentation of Fraction 3, where Luna alerted to peanut butter, a distractor, included in the round. The peanut butter was presented during the blank round, where Luna did not alert. Also, Luna had not previously alerted to peanut butter any other time it was presented during the trial.

**Table 5:** Number of correct responses to 20% photo-oxidized Hibernia crude oil, positive control and fractions, by each canine. An alert is represented by A and no alert/ response is represented by N. Within this session, 41 negative controls and distractors were presented. n represents the number of times a sample was presented throughout the trial.

	20% Photo-oxidized Hibernia Crude Oil				
	Positive Control (n = 4)	F 1 (n = 1)	F 2 (n = 1)	F 3 (n = 1)	False Response (n = 41)
**Poppy**	A	A	A	A	0
**Nika**	A	A	A	A	0
**Luna**	A*	A	A	A*	1
**Total Response**	**12**	**3**	**3**	**3**	**1 out of 41**
**Percent Alert**	**100%**	**100%**	**100%**	**100%**	**2.44%**

* Note: Handlers and observers of the session noticed Luna was hesitant on both the weathered Hibernia positive controls and fractions.

### Background threshold study

Canines were tested to see if they could distinguish the crude oil odor when additional non-target odors were added to the scent profile. When in the field, canines will experience a variety of background and non-target odors with the target odor adding another complexity to detection. As such it is important for canines to discriminate between the trained odor and to any background odors. To further explore this, a single SPME fiber was used to collect a mixture of the crude oil and a non-target odor. The SPME fiber was first exposed to the headspace of the WTI crude oil, followed by a 10 second exposure to a single non-target odor, isoamyl salicylate. The SPME fiber containing the mixture was inserted into the GC-F and the whole odor profile was collected onto a filter paper. This was repeated, with the single odor being replaced for a complex non-target odor, cologne (Versace eau fraiche). The results of the canine tests are provided in Table 6. Since the crude oil used in this portion of the study was the WTI crude oil, positive control 1 and 2 (Table 2) were used. Each of the mixtures were presented once, and the canines alerted to the mixtures 100% of the time, demonstrating the ability to ignore an additional odor and recognize the target odor.

**Table 6:** Number of correct responses to positive controls, single odor mixture, and complex odor mixture by each canine. An alert is represented by A and no alert/ response is represented by N. Within this session, 21 negative controls and distractors were presented. n represents the number of times a sample was presented throughout the trial.

	Positive Control (n = 4)	Single Odor (n = 1)	Complex Odor (n = 1)	False Response (n = 21)
**Poppy**	A	A	A	0
**Nika**	A	A	A	0
**Luna**	A	A	A	0
**Total Response**	**12**	**3**	**3**	**0**
**Percent Alert**	**100%**	**100%**	**100%**	**0%**

**Fig 4:**
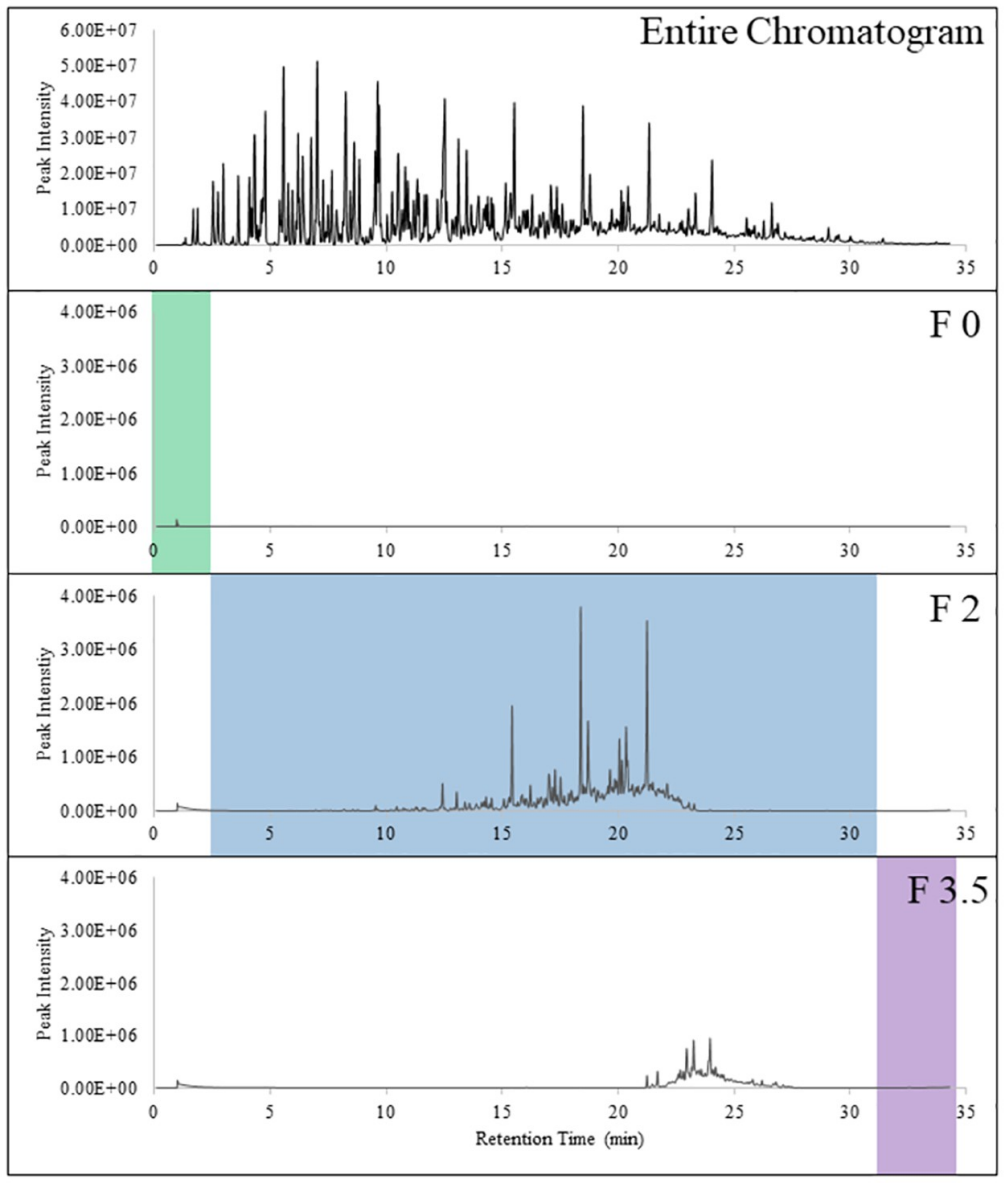
Limitation fractions used for trial 3. F0 was the highly volatile fraction collected between 0 to 2.5 min, F2 was the volatile fraction collected between 2.6 min to 29.59 min, and F3.5 was the semi-volatile fraction collected between 30 min to 34.29 min.

The final trial further explored the limitations the canine’s crude oil detection. Examining the entire odor chromogram, between r_t_ 0 to 2.5 min and r_t_ 30 to 34.29 min, there appears to be little to no VOCs present. The absence of VOCs means that they were below the limit of detection of the analytical instrument being used and as a result does not negate the presence of odor possible for the canine to detect. Hence, it was possible that the canines would be able to detect odors at those retention times though no VOCs were seen in the chromatogram. The new fractions were labeled as Fraction 0 (0–2.5 min) and Fraction 3.5 (30–34.29 min) and Fig 4 displays the chromatograms of these resulting profiles. Here, Fraction 2 profile was now collected between rt = 2.51 to 29.59 min and was used as a positive control. Results from the new fractions are provided in Table 7. Only two canines, Poppy and Nika, participated in this trial. The samples were presented a total of two times, for each sample, and the positive control was presented a total of six times. The canines alerted to Fraction 0, 25% of the time and Fraction 3.5, 80% of the time. The canines alerted 100% of the time to the positive control and did not alert to any distractors or negative controls. The canines failing to alert at Fraction 0, shows there are limitations at which the canines can detect crude oil.

**Table 7:** Number of correct responses to positive control and new fractions, by each canine. An alert is represented by A and no alert/ response is represented by N. Within this session, 56 negative controls and distractors were presented. n represents the number of times a sample was presented throughout the trial.

	Positive Controls (n = 6)	F 0 (n = 2)	F 3.5 (n = 2,3*)	False Response (n = 56)
**Poppy**	A	N	N, A*	0
**Nika**	A	A	A	0
**Total Response**	**12**	**1**	**5**	**0 out of 56**
**Percent Alert**	**100%**	**25%**	**80%**	**0%**

* Poppy had no alert on the first run through of Fraction 3.5. After Nika run the line, Poppy was re-run through the round and alerted on the fraction.

## Discussion

### Instrument limitations

There were limitations with the GC-F method. The end of the column releasing the eluent onto the filter paper was not heated (see Fig 1). The eluent flowing through the column would suddenly be cooled down from the internal temperature of the oven to room temperature, causing a delay in when the compounds would elute. The delay in the eluent caused there to be carry-over during the double collection of the fractions allowing for contamination to occur within Fraction 1 when peaks from Fraction 3 were noted. The issue was remediated by adding a cleaning method in between sample collection runs to ensure any compounds left behind in the column from the previous run would be eluted out. However, the issue could not be completely resolved, like in the case of Fraction 1 of the weathered Hibernia crude oil (see Fig 3) and with Fraction 3.5 (see Fig 4), as compounds from the semi-volatile fraction were present. For future studies, the instrument should be configured to include a heating element at the end of the column to ensure proper elution of the odor.

### Fresh crude oil

The canines were observed to recognize all three portions of the crude oil odor profile as well as generalize between differently sourced crude oils. Crude oil originates primarily from the remains of archaic organisms buried in ancient mud of swamps, lakes, and oceans. The chemical composition of crude oil will change dependent on the original composition of the organic matter, the environment, and the heat and time the oil was exposed to [30]. The oils are then classified based on the bulk properties, such as API gravity, density, viscosity, etc. as well as the elemental composition of the oil (high hydrogen or high carbon) to define the oil as light, medium, and heavy. Oil can also be classified based on sulfur content, making an oil with a higher sulfur content sour and a lower sulfur content sweet. Though, source location plays a role in the ratios of the hydrocarbons present, crude oils are relatively composed of the same four types of compounds: saturated hydrocarbons, aromatics, resins, and asphaltenes [31]. In the experiment, two differently sourced crude oils were presented as test probes. WTI and Hibernia crude oil are both classified as light and sweet crude oils, meaning they contain a higher ratio of saturated alkanes with a low sulfur content. All canines were previously trained on WTI crude oil and Bunker C crude, a heavy fuel oil, and two of the three canines (Poppy and Nina) were trained on highly weathered oil from the local beaches in Texas.

The results aligned with the conclusions in Vaughan et al, in that canines recognized and alerted to all three fractions in at least two of the three trials held. Vaughan et al. noted the canines seemed to recognize the semi-volatile fractions more readily than the highly volatile and volatile fractions [22], whereas in this study, all three fractions were detected at equal rates. “Furthermore, the canines alerted to both sets of fractions 100% from the fresh WTI and the fresh Hibernia crude oils (Tables 3 and 4), demonstrating their ability to generalize between differently sourced oils.” Considering the nature of the oils to which the canines were previously trained, it is clear that these canines generalize amongst crude oils ignoring the differences between oils and thus did not hesitate when two distinct oils were presented. Further studies could be conducted to train canines to discriminate between differently sourced crude oils based on their training materials.

### Weathered crude oil

The results from the weathered crude oil showed the canines were able to recognize to all three fractions of the odor profile. “Weathered” is an all-encompassing term for the physical, chemical, and biological processes crude oil can undergo after a spill. Weathering includes evaporation, emulsification, natural dispersion, dissolution, biodegradation, photo-oxidation, and sedimentation [31]. Evaporation and dissolution are two of the first weathering processes to physical change the crude oil causing the loss of the lighter hydrocarbons and aromatics, leaving the heavier volatiles and semi-volatiles [32]. Photo-oxidation can also occur, chemically changing the oil through the formation of oxygenated compounds [32], changing the odor profile of the oil. The Hibernia crude oil had been weathered using an enclosed wave pool and solar source, to perform photooxidation. Fig 3 shows the loss of the highly volatile fraction and a lower abundance of compounds within the volatile fraction due to the subsequent weathering it had undergone. This is further shown by the comparison of the tentatively identified compounds in S1 and S2 Tables. The 20% photo-oxidized Hibernia has considerably less compounds within fractions 1 and 2 than the fresh WTI, and a noticeable decrease in abundance, noted by the area %. Even with the loss of the highly volatile compounds, the canines alerted to the weathered fractions 100% of the time (Table 5). For Fraction 1, this indicates that a low, undetectable amount of odor per the GC-MS, was present on the filter paper, or the canines were alerting to the carry-over described in the limitation section of the results. Further testing would need to be conducted to better draw a conclusion.

Canines alerted 100% to the volatiles and semi-volatiles showing these fractions had not been substantially alter by the weathering to cause for no response. Again, the canines were trained on a light and sweet oil, residual oil, and weathered oil, so it is likely the canines were familiar and generalized in training to identify the weathered fraction testing probes. It was noted by the blind observer that, throughout the weathered fraction testing, Luna hesitated when alerting to both the positive controls and to the fractions. Luna had previously been trained only on fresh crude oil, including both the WTI and Bunker C; however as discussed above, Bunker C is a heavy fuel oil meaning it is the heavy residual part of a crude oil after it has undergone refinement. These oils are often blended with a light crude oil, making their composition vary between batches. The composition of a Bunker C will contain a mixture of saturated, aromatic, and olefinic (alkenes) hydrocarbons, with a predominate carbon numbers of C_9_ to C_50_ [30]. This means the headspace of a Bunker C oil would most likely have a lower abundance of highly volatile compounds and a higher abundance of volatile and semi-volatile compounds. Luna may have associated the trained odor with the weathered oil, causing generalization from what was learned in training to the testing probes. It was also noted that the oil may have not been weathered enough to discriminate it from the trained fresh oils. This hypothesis would agree with DeChant et al. [17] study where canines were trained to discriminate between fresh and highly weathered crude oil. Canines were trained on Bunker C oil and here were successful in discriminating between the fresh and highly weathered. Considering what DeChant et al concluded, it is possible the weathered oil odor profile may have a similar profile to that of the Bunker C oil, explaining why Poppy and Nika readily alerted to it and why Luna, hesitantly alerted to the fractions. Further testing with a wider variety of differently weathered oils could be used to determine at which point a fresh crude oil canine would no longer be able to detect a weathered crude oil.

### Background threshold study

Lastly, the limitations study was conducted to observe if there was a point in which the canines could no longer detect the crude oil. Up to this point, the canines had alerted 100% of the time to all of the fractions, both fresh and weathered, testing probes presented. Two separate limitations were applied. The first was to observe how the canines would react if additional odors were present in the headspace. Here, the crude oil odor was exposed to a single odor in the form of isoamyl salicylate, a floral-smelling odor, and to a complex odor, cologne. Even with additional odor, canines alerted to the mixtures. It was noted that during the presentation of Fraction 1 of the fresh Hibernia crude oil, Luna falsely alerted on the odor of cologne, which was being presented as a distractor. It was thought that Luna had associated the cologne odor with the odor of crude oil due to the order in which the testing probes were presented, where the mixture of crude oil and cologne was presented in a round before the distractor. The same distractor was presented in a later round, where she did not alert. Waggoner et al. conducted a study observing a canine’s performance when extraneous odors were presented with a target odor. The researchers observed the canines performance decreased as the concentration of the extraneous odor increased. They also observed canines had a more difficult time when the extraneous odor was complex. Canines had lower accuracy and higher false alerts with the addition of the complex odor, leading them to conclude that the canines may have been overwhelmed with the additional odors [33]. The findings here align with what Waggoner et al. observed, in that with the cologne, Luna had a false alert in possible associating the cologne odor with the crude oil. Since the single and complex odor here were only added in a short amount of time, further studies could be conducted on increasing the concentration of these odors to determine the accuracy of the canines.

The second part of the limitation study was to observe if there was a point in which the canines could no longer detect the crude oil. Looking at the entire odor profile of WTI crude oil (Fig 2), no peaks are visible between r_t_ = 0 and 2.5 min and peaks at low abundancy between r_t_ = 30 and 34.29 min. Again, this may either be due to the limitations of the sampling method, of the analytical method, and/or the peaks present between those retention times are below the detection limit of the instrument. Since there is a noticeable lack of peaks at these retention times, new fractions were made to determine if the canines would be able to recognize any odor at those retention times. Canines alerted to Fraction 0, 25% of the time and Fraction 3.5, 80% of the time (Table 6). The low alert rate at Fraction 0 may indicate that there was no odor present at the initial portion of the odor profile, and therefore would not make a suitable training aid for fresh crude oil detection canines. Compounds at this beginning retention time may include small alkanes (between C2 and C4). Fraction 3.5 had a higher alert rate, though this may be due to the presence of semi-volatile fraction between r_t_ = 25 and 30 min. Even with the contamination from the semi-volatile portion, the fraction is different from the original Fraction 3 and the canine alert rate does show canines recognize the semi-volatile portion of the odor profile, further supporting canines overall generalization of crude oil between all the fractions.

## Conclusion

The study endeavored to better understand how canines process complex mixtures as well as to observe how canines generalize to complex mixtures. In order to do this a method was created that broke down the odor profile of crude oil into different fractions and was presented to trained crude oil detection canines to observe what groups of groups of odorants the canines alerted on. First, the canines were found to have a 100% response rate on all three fractions from both the fresh and weathered samples indicating that canines are capable of detecting crude oil from any fraction of the odor profile. Second, canines alerted 100% to fractions from two differently sourced fresh crude oils; one in which they had previously trained on (WTI) and another they had not been exposed to (Hibernia). This demonstrated their potential to generalize to any sourced fresh crude oil. Third, the fresh crude oil detection dog, Luna, was observed to hesitate around the weathered crude oil before alerting demonstrating a possible overlap between the weathered crude oil and the crude oils she was trained on causing her to generalize between the two oil. Future studies need to be conducted to determine where a fresh crude oil detection canine can no longer detect a weathered crude oil. Finally, the background threshold studies showed canines were able to detect crude oil when additional odors were added to the profiles as well as the canines had lower alert rate at the end fractions (Fraction 0 and 3.5). The overall results indicated that canines are capable of detecting crude oil from any fraction of the odor profile demonstrating the potential for a canine to generalize across a variety of crude oils and stages of weathering.

## Supporting information

S1 Table List of tentatively identified compounds from the entire headspace profile of fresh WTI. The compounds are highlighted in the color corresponding with its fraction found in Fig 2.
(PDF)

S2 Table List of tentatively identified compounds from the entire headspace profile of 20% photo-oxidized Hibernia crude oil. The compounds are highlighted in the color corresponding with its fraction found in Fig 3.
(PDF)
