# Supplementary material for: Exploring canine olfactory generalization using odor profile fractions from native crude oils
Source: PLoS One. 2024 Oct 17;19(10):e0311818. doi: 10.1371/journal.pone.0311818 (PMC11486409; doi:10.1371/journal.pone.0311818)
Supplement: S1 Table — The compounds are highlighted in the color corresponding with its fraction found in Fig 2. (PDF) [file pone.0311818.s001.pdf]

S1 Table: List of tentatively identified compounds from the entire headspace profile of fresh WTI. The compounds are highlighted in the color corresponding with its fraction found in Fig 2.

RT (min)	Compound	Area %
1.126	Propane	0.004
1.230	Isobutane	0.015
1.329	Butane	0.052
1.663	2-methyl butane	0.281
1.844	Pentane	0.286
2.143	2,2-dimethyl butane	0.044
2.517	2-methyl pentane	0.673
2.726	3-methyl pentane	0.613
2.956	n-Hexane	0.745
3.138	1-hexene	0.019
3.292	2,2-dimethyl pentane	0.042
3.379	2,4-dimethyl pentane	0.092
3.603	Methyl-cyclopentane	0.654
3.908	3,3-dimethyl pentane	0.043
4.083	2-methyl hexane	0.612
4.179	2,3-dimethyl pentane	0.358
4.301	Cyclohexane	1.654
4.518	3-ethyl pentane	0.139
4.591	trans-1,3-dimethyl cyclopentane	0.390
4.661	cis-1,3-dimethyl cyclopentane	0.441
4.769	Heptane	1.723
5.032	2-Heptene	0.017
5.170	2,2,3,3-tetramethyl butane	0.034
5.450	2,4-dimethyl hexane	0.200
5.664	3,3-dimethyl hexane	0.079
5.756	1,2,4-trimethyl cyclopentane	0.618
5.944	1,2,3-trimethyl cyclopentane	0.503
6.106	2,3-dimethyl hexane	0.208
6.182	2-methyl heptane	1.052
6.232	4-methyl heptane	0.303
6.388	3-methyl heptane	1.117
6.612	2-methyl-4-methylene hexane	0.058
6.746	1,3-dimethyl cyclohexane	1.262
6.937	Cis-1-ethyl-3-methyl-cyclopentane	0.163
7.005	Octane	2.171
7.159	1-ethyl-1-methyl-cyclopentane	0.094
7.267	1,2-dimethyl cyclohexane	0.547
7.385	2,3,5-trimethyl hexane	0.158
7.473	1,4-dimethyl cyclohexane	0.431

7.641	2,6-dimethyl heptane	0.774
7.844	2,5-dimethyl heptane	0.492
8.001	1,2,4-trimethyl cyclohexane	0.163
8.117	Propyl-cyclopentane	0.200
8.235	Ethyl-cyclohexane	2.191
8.605	2-methyl octane	1.305
8.704	1,3,5-trimethyl cyclohexane	0.020
8.824	3-methyl octane	0.876
8.926	1-methyl-3-(1-methylethyl)-cyclopentane	0.042
9.295	1,2,3-trimethyl cyclohexane	0.223
9.508	Ethylbenzene	1.239
9.613	Nonane	1.743
9.686	(m- and p-) Xylene	1.305
9.756	Methyl-cyclooctane	0.230
9.877	3,5-dimethyl octane	0.089
10.024	1,1,3,5-tetramethyl cyclohexane	0.335
10.239	1-Ethyl-4-methylcyclohexane	0.651
10.497	2,6-dimethyl octane	1.446
10.802	2-nonen-1-ol	0.896
10.916	Propyl-cyclohexane	0.727
11.165	4-ethyl octane	0.632
11.328	4-methyl nonane	0.897
11.398	2-methyl nonane	0.432
11.491	1-ethyl-2,3-dimethyl-cyclohexane	0.045
11.552	3-ethyl octane	0.117
11.632	3-methyl nonane	0.482
11.735	1,1,2,3-tetramethyl cyclohexane	0.664
11.978	trans-octahydro-1H-Indene	0.107
12.369	Methyl-cycloheptane	0.773
12.505	Decane	2.675
12.588	1,3,5-trimethyl benzene	0.557
12.832	2-ethyl-1,3-dimethyl cyclohexane	0.419
13.101	4-methyl decane	1.259
13.297	2,3,6,7-tetramethyl-octane	0.340
13.454	1,2,4-trimethyl benzene	1.149
13.649	5-(1-methylpropyl)-nonane	0.704
13.964	(2-methylpropyl)-cyclohexane	1.150
14.176	5-methyl decane	0.755
14.277	4-methyl decane	0.449
14.366	2-methyl decane	0.493
14.437	1-Ethyl-2,2,6-trimethylcyclohexane	0.174
14.515	1,2,3-trimethyl benzene	0.482

14.601	3-methyl decane	0.490
15.134	1-methyl-3-propyl-benzene	1.093
15.246	1-methyl-4-propyl-benzene	0.229
15.347	1-ethyl-3,5-dimethyl-benzene	0.820
15.506	Undecane	1.967
15.724	1-methyl-2-propyl benzene	0.328
15.891	2,6-dimethyl decane	0.785
16.007	4-ethyl-1,2-dimethyl benzene	0.352
16.083	2-ethyl-1,4-dimethyl benzene	0.568
16.278	1-ethyl,2,4-dimethyl benzene	0.679
16.452	trans-2-undecen-1-ol	0.395
16.753	2-methyl-trans-decalin	0.741
17.233	1,2,4,5-tetramethyl benzene	0.446
17.342	2-methy undecane	0.650
17.431	1,2,3,5-tetramethyl benzene	0.367
17.578	3-methyl undecane	0.636
17.740	1-methyl-3-(1-methylpropyl)-benzene	0.386
17.931	1-methyl-4-(1-methylpropyl)-benzene	0.347
18.051	(1,1-dimethyl propyl) benzene	0.524
18.467	Dodecane	1.944
18.558	1,2,3,4-tetramethyl benzene	0.367
18.771	2,6-dimethyl undecane	1.312
19.033	2-phenyl-dec-2-yl ester buyric acid	0.374
19.812	1-(2,4-dimethylphenyl)-1-propanone	0.304
19.915	1-ethyl-2,3-dihydro-1-methyl-1H-Indene	0.312
19.994	2-ethenyl-1,3,5 benzene	0.388
20.118	Cyclododecane	0.609
20.231	2-methyl dodecane	0.576
20.408	6,6-dimethyl undecane	0.856
20.468	3,9-dimethyl undecane	0.394
21.319	Tridecane	1.648
21.469	Cyclotetradecane	0.355
21.546	1,4-dimethyl-2-(2-methylpropyl) benzene	0.597
21.758	6-methyl tridecane	0.542
22.003	(1,2-dimethyl-1-propenyl)-benzene	0.333
22.179	1,2,3,4-tetrahydro-6-methyl-naphthalene	0.417
22.743	1-(1-methylethenyl)-2-(1-methylethyl)-benzene	0.387
22.877	4-methyl tridecane	0.232
23.012	Cyclotridecane	0.599
23.115	1,2,3,4-tetrahydro-5-methyl-naphthalene	0.230
23.232	3-methyl tridecane	0.226
23.318	2-methyl naphthalene	0.850
23.648	Cyclohexyl-benzene	0.219

24.033	Tetradecane	1.320
24.612	1,2,3,4-tetrahydro-1,1,6-trimethyl-naphthalene	0.209
25.524	2,6,10-trimethyl tridecane	0.383
25.632	4-(2-butenyl)-1,2-dimethyl benzene	0.216
25.744	Biphenyl	0.212
26.040	1-ethyl naphthalene	0.353
26.263	2,3-dimethyl naphthalene	0.301
26.607	Pentadecane	0.634
26.783	2,6-dimethyl naphthalene	0.323
26.870	1,4-dimethyl naphthalene	0.381
27.177	Diphenylmethane	0.227
27.304	2,7-dimethyl naphthalene	0.112
27.431	1,6-dimethyl naphthalene	0.156
27.536	1,7-dimethyl naphthalene	0.150
27.864	1,5-dimethyl naphthalene	0.144
28.422	2-methyl-1,1'-biphenyl	0.112
28.670	4-methyl-1,1'-biphenyl	0.119
28.789	2-methyl-1-propyl-naphthalene	0.109
29.055	Hexadecane	0.311
29.198	1-(2,2-Dimethylcyclopropyl)-2-phenylacetylene	0.085
29.375	1,6,7-trimethyl naphthalene	0.125
29.491	2,3,6-trimethyl naphthalene	0.172
29.703	2,2'-dimethylbiphenyl	0.043
29.809	1-methyl-3-(phenylmethyl) benzene	0.039
29.888	2,3,7-trimethyl benzo[b]thiophene	0.052
29.999	1,4,5-trimethyl naphthalene	0.113
30.080	2,6,10-trimethyl pentadecane	0.080
30.428	4,6,8-trimethylazulene	0.058
30.537	1,4,6-trimethyl naphthalene	0.069
30.917	4,4'-dimethyl biphenyl	0.031
31.028	3-(2-methyl-propenyl)-1H-indene	0.047
31.218	2,3,5-trimethyl naphthalene	0.047
31.407	Heptadecane	0.098
31.643	2-ethyl-5,7-dimethyl-Benzo[b]thiophene	0.020
31.732	3,5-dimethyl-1-(phenylmethyl)-benzene	0.019
31.806	Chamazulene	0.018
31.904	1,4,5,8-Tetramethylnaphthalene	0.016
32.017	1-ethyl-3,5-diisopropyl-benzene	0.009
32.188	9H-Fluoren-9-ol	0.031
32.530	1,2,3,4-tetramethyl naphthalene	0.020
33.022	4-methyl-dibenzofuran	0.010
33.099	1,6-Dimethyl-4-ethylnaphthalene	0.011
33.449	(4,5,5-trimethyl-1,3-cyclopentadien-1-yl)-benzene	0.006

33.507	1-methyl-7-(1-methylethyl) naphthalene	0.009
33.594	1,6-Dimethyl- 3-ethylnaphthalene	0.012
33.694	Octadecane	0.018
33.750	2,6,10,14-tetramethyl hexadecane	0.015
33.941	2-methyl-9H-Fluorene	0.007
34.070	4-methyl-9H-Fluorene	0.011
