# Supplementary material for: Exploring canine olfactory generalization using odor profile fractions from native crude oils
Source: PLoS One. 2024 Oct 17;19(10):e0311818. doi: 10.1371/journal.pone.0311818 (PMC11486409; doi:10.1371/journal.pone.0311818)
Supplement: S2 Table — The compounds are highlighted in the color corresponding with its fraction found in Fig 3. (PDF) [file pone.0311818.s002.pdf]

S2 Table: List of tentatively identified compounds from the entire headspace profile of 20% photo-oxidized Hibernia crude oil. The compounds are highlighted in the color corresponding with its fraction found in Fig 3.

RT (min)	Compound	Area %
1.842	Pentane	0.001
2.516	2-methyl pentane	0.002
2.952	n-Hexane	0.006
3.598	Methyl-cyclopentane	0.004
4.077	2-methyl hexane	0.004
4.174	2,3-dimethyl pentane	0.001
4.306	Cyclohexane	0.010
4.585	1,3-dimethyl cyclopentane	0.002
4.640	Benzene	0.005
4.757	Heptane	0.023
5.438	2,4-dimethyl hexane	0.003
5.540	Methyl-cyclohexane	0.038
5.753	Ethyl-cyclopentane	0.009
5.929	1,2,3-trimethyl cyclopentane	0.008
6.096	3,4-diethyl hexane	0.005
6.169	2-methyl heptane	0.047
6.222	4-methyl heptane	0.015
6.376	3-methyl heptane	0.046
6.605	1,2,4-trimethyl cyclopentane	0.001
6.736	1,3-dimethyl cyclohexane	0.077
6.927	1-ethyl-3-methyl cyclopentane	0.008
6.990	Octane	0.422
7.171	1-ethyl-1-methyl cyclopentane	0.003
7.255	1,2-dimethyl cyclohexane	0.050
7.629	2,6-dimethyl heptane	0.107
7.833	2,5-dimethyl heptane	0.081
7.986	1,3,5-trimethyl cyclohexane	0.027
8.103	Propyl-cyclopentane	0.055
8.218	Ethyl-cyclohexane	0.442
8.593	2,4-dimethyl heptane	0.529
8.693	1,1,2-trimethyl cyclohexane	0.004
8.810	3-methyl octane	0.322
8.920	1-methyl-3-(1-methylethyl)-cyclopentane	0.009
8.986	Methyl-cycloheptane	0.017
9.177	(2-methylpropyl) cyclopentane	0.030
9.496	Ethylbenzene	0.504
9.588	Nonane	1.598
9.669	(m- and p-) Xylene	0.896

9.738	Methyl-cyclooctane	0.071
9.868	3,5-dimethyl octane	0.026
10.013	2,4,6-trimethyl heptane	0.199
10.228	1-ethyl-2-methyl cyclohexane	0.392
10.324	2-propyl-1-heptanol	0.105
10.475	o-Xylene	1.030
10.789	2-nonen-1-ol	0.502
10.903	Propyl-cyclohexane	0.678
11.158	4-ethyl octane	0.348
11.319	4-methyl nonane	0.853
11.386	2-methyl nonane	0.575
11.481	2-ethyl-1,3-dimethyl cyclohexane	0.018
11.543	3-ethyl octane	0.060
11.620	3-methyl nonane	0.529
11.974	trans-octahydro-1H-Indene	0.111
12.182	Propyl benzene	0.688
12.354	1-methyl-3-(2-methylpropyl) cyclopentane	0.763
12.490	Decane	3.521
12.577	1,3,5-trimethyl benzene	0.433
12.826	5-methyl-2-(1-methyl)-1-hexanol	0.478
12.978	2-butyl-1-octanol	0.433
13.088	1-ethyl-2-methyl benzene	1.337
13.277	5-ethyl-2-methyl octane	0.355
13.441	1,2,4-trimethyl benzene	1.412
13.638	3,7-dimethyl nonane	0.570
13.957	Butyl-cyclohexane	1.371
14.165	5-methyl decane	0.931
14.265	4-methyl decane	0.765
14.356	2-methyl decane	0.804
14.423	Hexyl-cyclopentane	0.084
14.506	1,2,3-trimethyl benzene	0.702
14.591	3-methyl decane	0.727
15.127	1-methyl-3-propyl benzene	1.346
15.237	1-methyl-4-propyl benzene	0.265
15.310	n-butyl benzene	1.122
15.495	Undecane	3.560
15.600	1,2-diethyl benzene	0.437
15.717	1-methyl-2-propyl benzene	0.826
15.997	1-ethyl-2,3-dimethyl benzene	0.442
16.075	2-ethyl-1,3-dimethyl benzene	0.684
16.269	1-ethyl,2,4-dimethyl benzene	0.662
16.438	4-ethyl decane	0.559
16.710	o-cymene	0.839

17.223	4-methyl undecane	0.827
17.332	2-methyl undecane	1.086
17.421	1,2,3,5-tetramethyl benzene	0.414
17.569	3-methyl undecane	0.945
17.730	1-methyl-4-(1-methylpropyl)-benzene	0.498
17.925	1-methyl-3-(1-methylpropyl)-benzene	0.438
18.067	(1,1-dimethylpropyl) benzene	0.709
18.350	2-Methyl-7-exo-vinylbicyclo[4.2.0]oct-1(2)-ene	0.314
18.458	Dodecane	3.596
18.549	1,2,3,4-tetramethyl benzene	0.626
18.640	1-methyl-2-(1-methylpropyl)-benzene	0.464
18.749	2,6-dimethyl undecane	1.596
19.369	2,6,8-trimethyl decane	0.425
19.609	(1-methyl-1-butenyl)-benzene	0.508
19.693	2,3-dihydro-1,2-dimethyl-1H-Indene	0.582
19.831	7-ethyl-bicyclo[4.2.1]nona-2,4,7-triene	0.404
19.905	6-methyl dodecane	0.574
19.979	2,4-dimethyl undecane	0.679
20.109	Cyclododecane	1.102
20.215	Naphthalene	1.468
20.396	2,6,10-trimethyl dodecane	1.081
20.459	2,9-dimethyl undecane	0.746
20.594	Decahydro-2,6-dimethyl-naphthalene	0.226
21.308	Tridecane	2.958
21.537	1,4-dimethyl-2-(2-methylpropyl) benzene	0.442
21.997	(1,2-dimethyl-1-propenyl) benzene	0.242
22.167	1,2,3,4-tetrahydro-5-methyl-naphthalene	0.524
22.642	6-methyl tridecane	0.685
22.734	1,2,3,4-tetrahydro-5,7-dimethyl-naphthalene	0.568
22.870	4-methyl tridecane	0.458
23.008	Heptylcyclohexane/ 2,3-dimethyl dodecane	1.178
23.222	9-methyl tridecane	0.376
23.329	2-methyl naphthalene	1.692
23.640	5,6,7,8,9,10-Hexahydrobenzocyclooctene	0.393
23.806	1-(2-butenyl)-2,3-dimethyl-benzene	0.427
23.959	1-methyl naphthalene	0.956
24.020	Tetradecane	2.035
24.607	1,2,3,4-tetrahydro-1,1,6-trimethyl-naphthalene	0.485
24.895	1,2,3,4-tetrahydro-2,5,8-trimethyl-naphthalene	0.413
25.263	2,5-dimethyl tridecane	0.369
25.513	2,6,11-trimethyl dodecane	0.554
25.735	Biphenyl	0.351
26.023	1-ethyl naphthalene	0.360

26.108	2-methyl-1,1'-biphenyl	0.190
26.254	2,3-dimethyl naphthalene	0.625
26.598	Pentadecane	1.097
26.740	2,6-dimethyl naphthalene	0.665
26.860	1,4-dimethyl naphthalene	0.718
27.298	2,7-dimethyl naphthalene	0.216
27.423	1,6-dimethyl naphthalene	0.233
27.529	1,7-dimethyl naphthalene	0.280
27.762	6-methyl pentadecane	0.135
27.857	1,5-dimethyl naphthalene	0.236
28.004	4-methyl pentadecane	0.137
28.118	2-methyl pentadecane	0.185
28.415	1-propyl naphthalene	0.310
28.665	4-methyl-1,1'-biphenyl	0.189
28.782	2-(1-methylethyl) naphthalene	0.154
29.046	Hexadecane	0.497
29.190	5-Methyl-1-phenylhexa-1,3,4-triene	0.112
29.366	1,6,7-trimethyl naphthalene	0.203
29.483	2,3,6-trimethyl naphthalene	0.153
29.582	3-chloro-,5,6,7,8-tetrahydrocinnoline	0.131
29.990	1,4,5-trimethyl naphthalene	0.185
30.073	2,6,10-trimethyl pentadecane	0.078
30.150	1,2,3,4-tetramethyl naphthalene	0.037
30.418	4,6,8-trimethylazulene	0.116
30.528	1,4,6-trimethyl naphthalene	0.107
30.909	4,4'-dimethylbiphenyl	0.052
31.019	2-methyl-1-propyl naphthalene	0.069
31.209	2,3,5-trimethyl naphthalene	0.068
31.398	Heptadecane	0.206
31.627	1-(chloromethyl)-2-methyl-naphthalene	0.024
32.507	1,2-dihydro-1-methyl-1-acenaphthylenol	0.015
33.015	9H-Fluoren-9-ol	0.012
33.093	Chamazulene	0.011
33.501	1,6-dimethyl-3-ethyl naphthalene	0.008
33.689	Octadecane	0.045
33.744	2,6,10,14-tetramethyl hexadecane	0.030
33.870	1-methyl-9H-fluorene	0.008
33.936	2-methyl-9H-fluorene	0.011
34.066	4-methyl-9H-fluorene	0.019
34.147	Dehydrochamazulene	0.003
